# Transfer Learning-Based Integration of Dual Imaging Modalities for Enhanced Classification Accuracy in Confocal Laser Endomicroscopy of Lung Cancer

**DOI:** 10.3390/cancers17040611

**Published:** 2025-02-11

**Authors:** Mircea-Sebastian Șerbănescu, Liliana Streba, Alin Dragoș Demetrian, Andreea-Georgiana Gheorghe, Mădălin Mămuleanu, Daniel-Nicolae Pirici, Costin-Teodor Streba

**Affiliations:** 1 Department of Medical Informatics and Statistics, University of Medicine and Pharmacy of Craiova, 200349 Craiova, Romania; 2 Department of Oncology and Palliative Care, University of Medicine and Pharmacy of Craiova, 200349 Craiova, Romania; 3 Department of Thoracic Surgery, University of Medicine and Pharmacy of Craiova, 200349 Craiova, Romania; 4 Doctoral School, University of Medicine and Pharmacy of Craiova, 200349 Craiova, Romania; 5 Department of Automatic Control and Electronics, University of Craiova, 200585 Craiova, Romania; 6 Department of Histology, University of Medicine and Pharmacy of Craiova, 200349 Craiova, Romania; 7 Department of Pulmonology, University of Medicine and Pharmacy of Craiova, 200349 Craiova, Romania

**Keywords:** lung cancer, confocal laser endomicroscopy, transfer learning, deep learning, histological imaging, dual transfer learning, classification accuracy, medical imaging, multi-modal integration

## Abstract

Background/Objectives: Lung cancer remains the leading cause of cancer-related mortality, underscoring the need for improved diagnostic methods. This study seeks to enhance the classification accuracy of confocal laser endomicroscopy (pCLE) images for lung cancer by applying a dual transfer learning (TL) approach that incorporates histological imaging data. Methods: Histological samples and pCLE images, collected from 40 patients undergoing curative lung cancer surgeries, were selected to create 2 balanced datasets (800 benign and 800 malignant images each). Three CNN architectures—AlexNet, GoogLeNet, and ResNet—were pre-trained on ImageNet and re-trained on pCLE images (confocal TL) or using dual TL (first re-trained on histological images, then pCLE). Model performance was evaluated using accuracy and AUC across 50 independent runs with 10-fold cross-validation. Results: The dual TL approach statistically significant outperformed confocal TL, with AlexNet achieving a mean accuracy of 94.97% and an AUC of 0.98, surpassing GoogLeNet (91.43% accuracy, 0.97 AUC) and ResNet (89.87% accuracy, 0.96 AUC). All networks demonstrated statistically significant (*p* < 0.001) improvements in performance with dual TL. Additionally, dual TL models showed reductions in both false positives and false negatives, with class activation mappings highlighting enhanced focus on diagnostically relevant regions. Conclusions: Dual TL, integrating histological and pCLE imaging, results in a statistically significant improvement in lung cancer classification. This approach offers a promising framework for enhanced tissue classification. and with future development and testing, iy has the potential to improve patient outcomes.

## Simple Summary

Lung cancer is the most common and deadly cancer worldwide, making early and accurate diagnosis essential for improving patient outcomes. Traditional diagnostic methods often require invasive biopsies, which can be uncomfortable and carry risks. This study investigates a new approach that combines two imaging techniques, namely confocal laser endomicroscopy (pCLE), which provides real-time microscopic views of lung tissue, and standard histological analysis of stained tissue samples. By using a machine learning method called dual transfer learning, we trained computer models to better differentiate between cancerous and non-cancerous tissues in pCLE. Our findings demonstrate that integrating these two imaging modalities results in a statistically significant enhancement in the accuracy of lung cancer detection. This innovative approach has the potential to make diagnoses faster and less invasive, ultimately leading to better patient care and more effective treatment strategies.

## 1. Introduction

Lung cancer remains one of the most significant medical challenges worldwide, both in terms of prevalence and associated mortality. Epidemiological studies indicate that this pathology ranks first among cancer-related deaths globally, with a markedly increased incidence in countries with high Human Development Index scores, where smoking and prolonged exposure to risk factors result in alarmingly high mortality rates [1]. Furthermore, a notable sex disparity persists, with men being more affected, although recent years have shown a rising incidence among women [1]. Countries undergoing economic transition are also experiencing an increase in lung cancer incidence due to air pollution and occupational exposure to toxic agents [2]. Unfortunately, the majority of cases are diagnosed at advanced stages, which is reflected in a 5-year survival rate below 20% [3].

The risk factors for lung cancer are well documented, with smoking directly associated with the largest proportion of cases [4]. Air pollution, particularly through the inhalation of fine particles (PM2.5) and chemical agents, such as nitrogen dioxide, is another critical contributor to incidence [5]. Additionally, occupational exposure to hazardous substances, such as asbestos, significantly increases oncological risk [6]. The socioeconomic consequences are considerable, encompassing both direct costs (treatment, hospitalization, advanced therapies) and indirect costs (productivity loss, family impact), while early screening and diagnostic methods, such as low-dose computed tomography (LDCT), generate additional financial burdens [7]. In resource-limited economies, restricted access to early detection programs exacerbates the disease burden [8].

In this context, improving diagnostic methods becomes essential. LDCT screening has proven effective for early detection, particularly in individuals with elevated risk factors [9]. Moreover, the introduction of molecular biomarkers and complementary analyses offers a promising perspective for earlier diagnosis [10], especially when targeted at high-risk populations [11]. However, challenges related to the specificity and sensitivity of conventional methods remain.

A complementary and increasingly utilized approach in bronchoscopy is probe-based confocal laser endomicroscopy (pCLE). This technique enables real-time in vivo acquisition of microscopic images of pulmonary tissue, providing valuable insights into cellular architecture and structural abnormalities [12]. Explorations, like pCLE, can differentiate neoplastic from normal tissue, optimizing biopsy sampling by guiding toward highly suspicious areas [13,14]. While the technique does not entirely replace biopsy—as tumors lack autofluorescence and require the use of contrast agents—pCLE significantly accelerates the diagnostic process [15]. Features, such as cellular atypia, disorganized elastic fibers, and alterations in alveolar structure, can be rapidly identified [16]. The sensitivity and specificity of pCLE for certain pathologies, such as *Pneumocystis jirovecii* pneumonia, are notably high, surpassing conventional methods [17]. Additionally, this technique is valuable in monitoring post-lung transplant patients by detecting signs of acute rejection [18]. The development of standardized interpretation guidelines and specialist training ensures high diagnostic accuracy [19,20].

Alongside these imaging advances, artificial intelligence (AI), particularly deep learning (DL), has gained prominence in medical image diagnostics. Deep convolutional neural network (CNN) architectures, such as ResNet, VGG, and Inception, have demonstrated remarkable performance in classifying pulmonary nodules on radiographs and CT scans [21,22]. In histopathology, DL can uncover subtle patterns and microscopic features that are challenging for human experts to detect [23]. Nevertheless, a major limitation in the medical field remains the lack of sufficiently labeled data. To overcome this challenge, transfer learning (TL) allows for the reuse of knowledge acquired by networks initially trained on massive datasets (e.g., ImageNet), adapting them to rarer and more specialized medical images [24,25]. It has been proven that TL thus reduces the time and costs associated with annotation while improving the models’ ability to generalize on limited datasets [26].

Pre-trained networks, such as AlexNet, GoogLeNet, and ResNet, have been successfully employed in classifying pCLE and histopathological images, achieving very high accuracy [27,28]. Furthermore, dual TL—integrating knowledge from two domains—can further enhance the performance of these models [29,30]. Multi-modal data integration (e.g., CT imaging, histopathology, biomarkers) represents another frontier in lung cancer diagnostics. Recent methods propose the fusion of information extracted from various sources, increasing the accuracy and robustness of diagnoses [31].

A notable example highlights the potential of combining pCLE images with other modalities to achieve superior detection and classification of pulmonary lesions [30]. The integration of histopathological data with pCLE imaging, coupled with TL techniques, can substantially improve the likelihood of precise and early diagnosis. This multi-modal approach addresses challenges related to incomplete, misaligned, or heterogeneous data, bringing us closer to an integrative framework for computer-assisted diagnostics.

This highlights the pressing need for substantial improvements in early screening and diagnostic methods to address the high incidence and mortality rates associated with lung cancer. Techniques, such as pCLE, have demonstrated utility in rapidly obtaining in vivo microscopic information, while AI, particularly DL and TL, opens new avenues by reducing reliance on large datasets and diversifying information sources. The multi-modal integration of imaging and clinical data, as illustrated by recent studies, represents a crucial step toward more accurate, personalized, and cost-effective diagnostics. Continuous development of these technologies and associated methodologies will play an essential role in improving the prognosis and quality of life for patients diagnosed with lung cancer.

Our research focuses on exploring the potential of dual TL by integrating two imaging modalities. The objective is to enhance the classification accuracy of pCLE for lung cancer images by introducing knowledge derived from histology techniques, thereby addressing the challenges associated with the clinical use of pCLE. This approach demonstrated the efficacy of leveraging multiple sources of information to improve diagnostic outcomes in lung cancer.

## 2. Materials and Methods

This study was conducted between January 2019 and January 2021 on a cohort of 40 patients from the Thoracic Surgery Clinic at the Emergency County Hospital of Craiova, Romania, who had undergone curative lung cancer surgeries. The target population comprised residents of Dolj County, Romania, with the only inclusion criterion being a recommendation for curative lung cancer surgery. Ethical approval was granted by the Ethics Committee of the University of Medicine and Pharmacy of Craiova, and informed consent was obtained from all participants.

Histological samples were obtained intraoperatively, labeled with acriflavine, and analyzed ex vivo using a pCLE probe (pCLE, CellVizio^®^, Mauna Kea Technologies, Paris, France). The resulting images were saved for subsequent analysis. The same tissue samples were processed histopathologically and stained with hematoxylin–eosin for tumor localization and silver impregnation, which followed standardized clinical staining protocols. Whole-slide scanning was performed to digitize the histology slides, and the images were stored electronically. The scanning process utilized a Nikon Eclipse 90i (Nikon Instruments Inc., Melville, NY, USA) motorized microscope equipped with a Prior OptiScan ES111 (Prior Scientific Inc., Rockland, MA, USA) motorized stage, a 16-megapixel Nikon DS-Ri-2 CMOS cooled camera (Nikon Instruments Inc., Melville, NY, USA), and Nikon NIS-Elements AR software (Nikon Instruments Inc., Melville, NY, USA) for image acquisition and analysis.

Two distinct datasets were obtained: one derived from the pCLE images (confocal dataset) and one from the histology slides (histology dataset).

**Figure 1:**
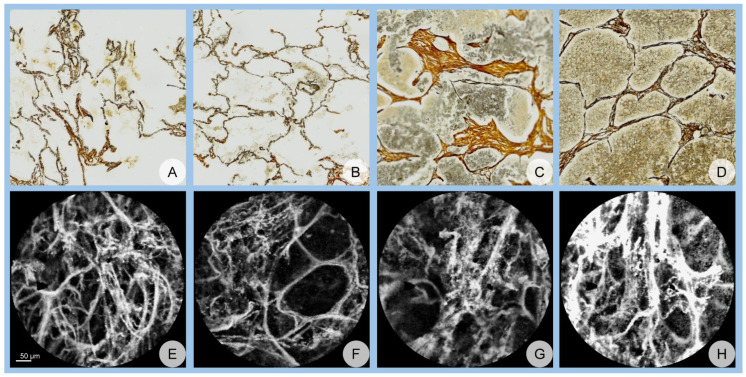
Representative samples from the dataset: (**A**–**D**) Images from the histology dataset, where (**A**,**B**) represent benign samples and (**C**,**D**) represent malignant samples. (**E**–**H**) Images from the confocal dataset, where (**E**,**F**) correspond to benign samples and (**G**,**H**) correspond to malignant samples.

Both datasets were independently reviewed by two pathologists, who annotated malignant and benign regions without access to any clinical information or diagnostic data. Hematoxylin–eosin-stained images were registered onto silver impregnation images to facilitate clear tumor identification. The resulting digital slides were divided into tiles of 512 × 512 pixels. These tiles were presented to the pathologists for annotation, and the labeled silver impregnation images were subsequently saved. Confocal images (512 × 512 pixels) were obtained from confocal video recordings, and these images were also labeled by the pathologists. From each patient, 10 image samples were selected for each class (malignant and benign) and for each imaging technique. Complete inter-observer agreement was required for all images. Images with discrepancies in labeling were replaced to ensure consistency. This resulted in a total of 800 confocal images and 800 histology images, equally balanced between malignant and benign labels. Representative examples of images from both datasets are illustrated in Figure 1.

The original dataset did not undergo normalization or noise reduction prior to analysis. However, data augmentation was applied during the training process using Matlab’s (The MathWorks, Inc., Natick, MA, USA) built-in image data augmenter. The following augmentation parameters were used: reflection along the X and Y axes, random rotations ranging from 0° to 360°, and scaling factors between 0.9 and 1.1.

The datasets were divided to facilitate a 10-fold cross-validation evaluation. Specifically, 70% of the data was allocated for training, 20% for validation, and 10% for testing. This split was determined prior to data augmentation, and the images were randomly selected before each network training iteration.

Given the inherent challenges in precise spatial registration between the two imaging modalities, we explored a dual TL approach to enhance the performance of the confocal image classifier. This approach leverages features learned from the histology dataset to improve classification accuracy on the confocal dataset.

Three DL networks—AlexNet [32], GoogLeNet [33], and ResNet [34]—were employed for this purpose. Several factors influenced the selection of these architectures. First, all three networks have pre-trained versions on ImageNet [35] available for Matlab, providing a solid foundation for TL. Second, given the relatively small size of our dataset, choosing more complex architectures with a greater number of trainable parameters could have led to overfitting, while these networks strike a balance between complexity and generalizability. Lastly, the team’s experience with these architectures and their proven success in other medical applications [27,36] further justified their selection for this task.

AlexNet, introduced by Krizhevsky et al. [37] in 2012, represents a seminal work in the field of DL, particularly in image classification tasks. It is a CNN architecture consisting of five convolutional layers, each followed by rectified linear unit activations to introduce non-linearity. These layers are interspersed with max-pooling layers to reduce spatial dimensions and improve computational efficiency. The network transitions to the fully connected layers with two fully connected layers, ultimately concluding with a 1000-way softmax layer to classify images into 1000 categories from the ImageNet dataset. AlexNet was among the first architectures to leverage dropout regularization to mitigate overfitting and data augmentation techniques, such as random cropping and flipping, to enhance model robustness. The model is freely available for download and compatible with Matlab.

GoogLeNet, introduced in 2014 by Szegedy et al. [38], marked a departure from traditional deep CNNs by employing a more sophisticated, non-linear architecture through the introduction of the Inception module. The network consists of 22 layers but is computationally efficient due to its modular design. The Inception module enables the simultaneous application of convolutions with multiple filter sizes (e.g., 1 × 1, 3 × 3, and 5 × 5) and max-pooling, with the results concatenated into a single output. This architecture reduces the number of parameters compared to earlier CNNs while preserving representational capacity. Key innovations in GoogLeNet include the use of global average pooling instead of fully connected layers at the network’s end, significantly reducing overfitting. Additionally, GoogLeNet incorporates auxiliary classifiers at intermediate layers to facilitate gradient flow during backpropagation and improve training stability. GoogLeNet’s model is freely available for download and compatible with Matlab.

The ResNet-18 architecture, introduced by He et al. [39] in 2016, is a part of the Residual Network (ResNet) family, which revolutionized DL by addressing the vanishing gradient problem in very deep networks. ResNet-18 features 18 layers organized into a series of residual blocks, where each block introduces skip connections (also called shortcuts) that bypass one or more layers. These skip connections allow the network to learn residual mappings rather than full transformations, facilitating the efficient training of deeper models. Each residual block typically consists of two convolutional layers with batch normalization and ReLU activations, followed by the addition of the input (identity) to the output. This approach significantly improves gradient flow during training and mitigates the degradation problem seen in deeper networks. The model is also freely available for download and compatible with Matlab.

Initially, the networks were pre-trained on the ImageNet dataset and re-trained for binary classification (malignant vs. benign) using the histology dataset. This constituted the first phase of TL. Subsequently, the resulting networks were re-trained on the confocal dataset without modifying the output layer, representing the second TL phase. The final models, therefore, integrated knowledge from both imaging techniques, creating a dual TL framework.

Each of these pre-trained network architectures was adapted to accommodate a new classification task distinct from their original purpose of classifying real-world images, such as those from the ImageNet dataset. Specifically, the original networks were modified to suit the requirements of a binary classification problem. The final fully connected layer and the output layer of each model were replaced to reflect the new dataset’s structure. The last fully connected layer was redesigned to output features corresponding to the binary classification problem, while the softmax output layer was modified to produce probabilities for two classes, aligning with the label structure of the dataset.

The training process for all three networks was conducted under identical conditions to ensure a fair comparison of their performance. The hyperparameters were set empirically based on preliminary experimentation. A mini-batch size of 20 was employed to achieve a balance between computational efficiency and the stability of gradient estimates. The initial learning rate was set to 0.0001 to ensure gradual convergence and to minimize the risk of overshooting during optimization. Furthermore, validation patience was configured to 4, meaning the training would halt early if the validation loss did not improve over four consecutive epochs, helping to prevent overfitting.

All networks were trained using stochastic gradient descent with momentum as the optimization algorithm. The momentum mechanism was incorporated to accelerate convergence by dampening oscillations in the optimization trajectory and to help escape shallow local minima. This consistent setup across the architectures ensured a standardized environment for evaluating the models on the new classification task, providing a reliable basis for comparative analysis.

The decision to utilize pre-trained DL networks rather than developing a custom mode was based on the size of the dataset, which is relatively small and insufficient for training a new network from scratch without risking overfitting. Pre-trained networks leverage TL, where pre-existing trained weights from large datasets are used to improve model performance on smaller datasets. This approach ensures more reliable results and mitigates the risk of overfitting, thereby making it a suitable choice for the scope of our study.

**Figure 2:**
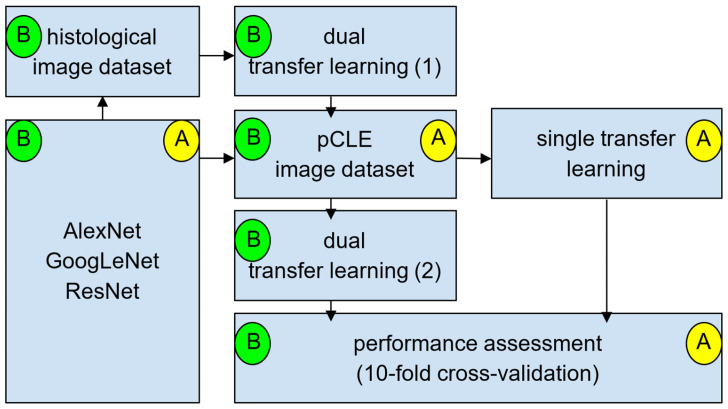
Study design schematic: The dual TL approach integrates features from histological and pCLE image datasets to improve classification accuracy. Path A (yellow) applies a single TL step directly on the pCLE image dataset. Path B (green) involves two sequential TL steps: the first is performed on the histological image dataset, followed by the second TL on the pCLE image dataset. The performance of both approaches is evaluated using a 10-fold cross-validation framework.

To benchmark the dual TL scenario, we conducted a control experiment wherein the same networks, initialized with ImageNet weights, were re-trained exclusively on the confocal dataset. This control experiment provided the confocal TL scenario. A graphical representation of the study design is presented in Figure 2.

To ensure robust and statistically reliable results, all DL models were executed independently over 50 iterations. The stochastic nature of DL algorithms necessitates multiple independent runs to adequately sample decision performance. For each run, a 10-fold cross-validation strategy was employed. At each iteration, 40 images of each class/scenario were randomly selected for testing prior to data augmentation.

Statistical analyses were conducted to confirm adequate power, with a two-tailed null hypothesis, a Type I error rate (α) of 0.05, and a target statistical power exceeding 95%.

To further elucidate the classification performance, confusion matrices were generated for two randomly selected networks from each scenario. These matrices illustrate the true versus predicted classifications for both benign and malignant classes.

To visualize and interpret the regions of the images that the networks focus on during classification, class activation mapping (CAM) was employed.

All DL model implementations, data processing, and statistical evaluations were performed using Matlab).

Computations were performed on an Intel^®^ Xeon^®^ Silver 4216 processor (Intel Corp., Santa Clara, CA, USA), equipped with 128 GB of RAM, and an NVIDIA^®^ Quadro^®^ RTX 6000 graphics processing unit (NVIDIA Corp., Santa Clara, CA, USA) with 24 GB of memory. The average training time for one network was 53 ± 13 min.

## 3. Results

A total of 50 DL networks were trained using the dual TL approach, integrating both histology and confocal datasets, and an additional 50 networks were trained exclusively on the confocal dataset for each of the 3 network architectures: AlexNet, GoogLeNet, and ResNet. The performance metrics, including accuracy and area under the curve (AUC), for both scenarios across all network architectures are summarized in Table 1 and Table 2, respectively.

### 3.1. Network Performance

#### 3.1.1. Accuracy Assessment

The dual TL scenario consistently outperformed the confocal TL scenario across all network architectures. Specifically, AlexNet achieved a mean accuracy of 94.97% (±1.76), GoogLeNet attained 91.43% (±2.17), and ResNet reached 89.87% (±2.15) in the dual TL scenario. In contrast, the confocal TL scenario yielded lower accuracies of 90.14% (±2.13) for AlexNet, 85.71% (±2.55) for GoogLeNet, and 84.65% (±1.84) for ResNet. An ANOVA test confirmed that these differences were statistically significant (*p* < 0.001). Furthermore, Student’s *t*-test revealed that the improvements in accuracy for the dual TL scenario were statistically significant across all network architectures (*p* < 0.001).

#### 3.1.2. AUC Assessment

Similarly, the AUC values demonstrated superior performance for the dual TL approach. AlexNet achieved an AUC of 0.98 (±0.01), GoogLeNet obtained 0.97 (±0.01), and ResNet reached 0.96 (±0.01) in the dual TL scenario. Comparatively, the confocal TL scenario resulted in AUCs of 0.97 (±0.01) for AlexNet, 0.93 (±0.02) for GoogLeNet, and 0.94 (±0.01) for ResNet (Table 1).

**Table 1:** Network performance accuracy (%) assessment.

Accuracy, Mean ± Standard Deviation (SD)	AlexNet	GoogLeNet	ResNet	ANOVA, *p*
Dual TL scenario	94.97 ± 1.76	91.43 ± 2.17	89.87 ± 2.15	<0.001
Confocal TL scenario	90.14 ± 2.13	85.71 ± 2.55	84.65 ± 1.84	<0.001
Student’s *t*-test, *p*	<0.001	<0.001	<0.001	

The ANOVA results indicated statistically significant differences between the two scenarios (*p* < 0.001), and Student’s *t*-test confirmed the statistical significance of these differences across all network architectures (*p* < 0.001) (Table 2).

**Table 2:** Network Performance AUC Assessment.

AUC, Mean ± Standard Deviation (SD)	AlexNet	GoogLeNet	ResNet	ANOVA, *p*
Dual TL scenario	0.98 ± 0.01	0.97 ± 0.01	0.96 ± 0.01	<0.001
Confocal TL scenario	0.97 ± 0.01	0.93 ± 0.02	0.94 ± 0.01	<0.001
Student’s *t*-test, *p*	<0.001	<0.001	<0.001	

### 3.2. Confusion Matrix Analysis

Table 3 presents the confusion matrices for AlexNet, GoogLeNet, and ResNet under both the confocal TL and dual TL scenarios.

In the confocal TL scenario, AlexNet correctly classified 98.75% of the benign and 89.25% of the malignant cases. GoogLeNet correctly identified 91.5% of the benign and 88.5% of the malignant cases. ResNet achieved correct classifications for 85.25% of the benign and 92% of the malignant cases.

Conversely, in the dual TL scenario, AlexNet demonstrated enhanced performance by correctly classifying 98.5% of the benign (a 0.25% decrease) and 94% of the malignant cases. GoogLeNet improved to 96.75% of the benign and 85.5% (a 3% decrease) of the malignant correctly classified cases. ResNet maintained a rate of 85.25% of the benign and improved to a rate of 93.25% for the malignant correctly classified cases.

These results indicate that the dual TL approach results in a statistically significant reduction in the number of misclassifications, particularly for malignant cases, thereby enhancing the diagnostic accuracy and reliability of the models (Table 3).

### 3.3. Class Activation Mapping

**Figure 3:**
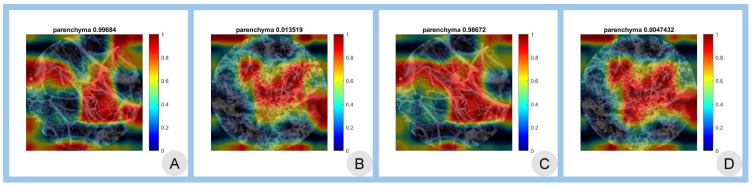
CAM visualization: Panels (**A**,**B**) depict CAMs for the confocal TL scenario, while panels (**C**,**D**) correspond to the dual TL scenario, both utilizing the best-performing AlexNet architecture. The highlighted regions indicate image areas that contribute to the classification decision, particularly fiber size and overlapping structures, offering insights into the model’s decision-making process.

Figure 3 illustrates the CAMs for both scenarios using the best-performing AlexNet architecture.

### 3.4. Summary of Performance Metrics

The comparative analysis of the network performances underscores the efficacy of the dual TL approach. Across all architectures, dual TL not only improved mean accuracy and AUC but also demonstrated greater consistency and reduced variability in performance metrics. The statistical analyses affirm that these improvements are not due to random chance, thereby validating the robustness and superiority of integrating dual imaging modalities through TL.

### 3.5. Statistical Significance

The comparative analysis between dual TL and confocal TL scenarios was rigorously evaluated using ANOVA and Student’s *t*-test, with all *p*-values reported as <0.001. This indicates a statistically significant improvement in both accuracy and AUC metrics when utilizing the dual TL approach across all network architectures. The substantial reduction in misclassifications, particularly in the malignant class, underscores the clinical relevance and potential of dual TL in enhancing diagnostic precision in lung cancer classification.

### 3.6. Overall Performance

The dual TL framework not only demonstrated higher accuracy and AUC but also exhibited greater consistency and reliability across multiple iterations. The reduced standard deviations in performance metrics suggest that dual TL mitigates the variability inherent in stochastic DL algorithms, thereby providing more stable and dependable diagnostic outputs.

The results highlight the superior performance of the dual TL approach in classifying lung cancer using pCLE and histological imaging. By leveraging complementary information from both imaging modalities, dual TL statistically significant enhances classification accuracy and AUC, offering a robust tool for early and precise lung cancer diagnosis.

## 4. Discussion

All resultant networks achieved accuracies and AUC values surpassing 80% and 0.8, respectively, indicating strong diagnostic capabilities. These results are broadly consistent with findings from other studies that reported comparable improvements in classification performance when applying TL to medical imaging tasks [40,41,42,43]. The influence of the chosen architecture remained statistically significant, with AlexNet yielding better outcomes than GoogLeNet and ResNet. This somewhat counterintuitive result, where a simpler architecture outperforms more complex ones, emphasizes the importance of careful design and optimization in TL workflows and resonates with observations made in other fields of medical image classification where architectural complexity does not always translate into superior results [44].

More importantly, regardless of the network architecture, the application of dual TL enhanced the classification accuracy by about 5%—a statistically significant improvement (*p* < 0.001)—and also increased the AUC by approximately 0.02. Similar improvements have been noted in other medical imaging contexts, where incorporating data from different sources or modalities contributed to better diagnostic metrics, as has been reported in various studies adopting TL to supplement limited domain-specific data [27]. Examination of the confusion matrices further underscored the benefits of dual TL, revealing fewer misclassifications for both benign and malignant categories. In particular, AlexNet exhibited a marked decrease in false benign predictions, while GoogLeNet, although showing a slight increase in false malignant predictions, reduced the false negative rate. These nuanced differences suggest that individual architectures may internalize the morphological cues provided by histology and pCLE to varying degrees, highlighting the value of combining complementary imaging data to fine-tune network decision boundaries.

The superior performance observed with dual TL was further supported by CAMs, which showed enlarged activation areas for both benign and malignant classes. By integrating histology-derived features into the learning process, the CNNs gained a more robust morphological foundation, enabling them to focus more accurately on diagnostically relevant regions within the pCLE images. The CAMs provide a clear view of how the two imaging modalities overlap, with class activations better highlighting fiber size/intersections. Such visualizations offer insight into the networks’ decision-making processes and illustrate how combining two imaging modalities enhances interpretability and reliability.

Our earlier research efforts have established that CNNs can accurately predict image diagnoses in various contexts, providing a strong basis for integrating multi-modal data [45,46,47,48]. While previous approaches often examined separate imaging modalities or made comparisons rather than integrations, the present dual TL approach leverages the synergy of histology and pCLE data to produce more refined diagnostic predictions. This integration aligns with prior explorations of tumor architecture, vascularization, and the interstitial fibrillary network using conventional and fractal dimension analyses [49,50,51,52,53]. The current findings reinforce those earlier conclusions, demonstrating that advanced DL frameworks can capture and extend these morphological correlations by combining the complementary strengths of histology and pCLE.

In the broader landscape of medical imaging, other researchers have consistently shown that carefully selected CNN architectures, like AlexNet, GoogLeNet, and ResNet, can effectively classify a range of pathologies once guided by TL [54,55,56,57,58]. More recent approaches have taken this further, using dual TL to integrate data from diverse sources and imaging modalities, thereby achieving remarkable accuracy and AUC values [59,60]. The improved metrics observed in this study reflect similar trends noted in multi-parametric imaging and radiomics-based analyses, which have successfully enhanced classification performance and mitigated the risk of overfitting [61,62,63,64,65,66].

The enhanced pCLE classifier could have direct clinical applications, allowing surgeons to apply the model’s output to resection specimens in real time. This would enable more accurate identification of clear resection margins, offering potential benefits in surgical outcomes.

### 4.1. Limitations

A primary limitation of this study lies in the reliance on relatively small and localized datasets. Although the dual TL approach substantially improved classification accuracy and AUC, these findings would benefit from confirmation on larger, more heterogeneous patient cohorts. Additionally, while the histology and pCLE imaging modalities provided complementary morphological cues, other data sources—such as molecular markers, clinical parameters, and advanced imaging sequences—could potentially further enhance diagnostic precision. The computational cost associated with repeatedly re-training CNNs remains another consideration, as does the inherent variability in histological interpretation and pCLE image acquisition. Future studies should also consider prospective, multi-center validations and incorporate interpretability strategies beyond CAM to better understand the underlying decision processes.

The results presented in Table 3 represent a randomly selected network from the 50 trained models. It is important to note that no specific constraints were imposed on the true positive or true negative rates for malignant cases during the training process. Consequently, the slight increase in false negatives observed in malignant classifications does not pose a significant concern. This is because the primary performance metric optimized during training was overall accuracy, which evaluates the model’s general classification performance across all classes. While false negatives are clinically relevant, their impact in this context is mitigated by the study’s focus on achieving high overall accuracy rather than specifically optimizing sensitivity or specificity for malignant cases. Future work could explore strategies, such as threshold adjustments or cost-sensitive learning, to further reduce false negatives without compromising accuracy.

Finally, the lack of standardized staining in the histological preparation may have an unknown impact on classification performance. It can be argued that the performance could improve with the use of standardized stain normalization. We previously proposed and validated a methodology for stain normalization [67], though it has not yet been applied clinically. Consequently, varying tissue preparation methods and staining techniques may potentially enhance classification performance.

### 4.2. Wrap-Up and Future Work

Worldwide, lung cancer remains the most common malignancy, with increasing incidence and mortality rates. Utilizing modern early diagnosis techniques is crucial as they may statistically significantly shorten the waiting time for histopathological results. The novel imaging technique—pCLE—has demonstrated its ability to accurately diagnose malignant lung lesions and with larger studies holds the potential to transform the role of the biopsy in lung endoscopy in the near future.

Future work should aim to address the current limitations by developing objective control mechanisms for histological staining processes to minimize variability. Additionally, efforts should be made to reduce the financial barriers associated with pCLE to facilitate its broader application and integration into routine diagnostic workflows. Expanding the dataset to include a larger and more diverse patient population will enhance the generalizability and robustness of the DL models. Furthermore, incorporating additional data modalities, such as molecular biomarkers and clinical parameters, could further improve diagnostic accuracy and model performance. Prospective, multi-center studies are also recommended to validate the TL approach across different clinical settings and populations, ensuring its applicability and scalability in real-world scenarios.

## 5. Conclusions

This study demonstrates that integrating histological image features through dual TL can enhance the diagnostic performance of CNN-based classification models applied to pCLE images in lung cancer. By incorporating complementary imaging modalities, the CNN models showed improvements in accuracy and AUC, with AlexNet achieving an increase in accuracy from 90.14 ± 2.13 to 94.97 ± 1.76, GoogLeNet from 85.71 ± 2.55 to 91.43 ± 2.17, and ResNet from 84.65 ± 1.84 to 89.87 ± 2.15. These results suggest that a multi-modal, TL-based approach may contribute to more reliable and interpretable diagnostic outputs, providing potential benefits for early and precise lung cancer detection.

However, the study has certain limitations that warrant further investigation. Specifically, the small dataset size, computational demands, and the lack of standardized histological staining protocols may affect the generalizability of the findings. Future work should address these limitations by incorporating larger, more diverse datasets and exploring methods to standardize preprocessing and staining. Ultimately, further studies are necessary to validate the proposed approach and assess its clinical utility in improving diagnostic outcomes.

## Figures and Tables

**Table 3:** Confusion matrices of selected networks in both scenarios.

			AlexNet		GoogLeNet		ResNet	
			Benign	Malignant	Benign	Malignant	Benign	Malignant
Actual class	Confocal TL scenario	Benign	395	43	366	46	341	32
		Malignant	5	357	34	354	59	368
	Dual TL scenario	Benign	394	24	387	58	341	27
		Malignant	6	376	13	342	59	373
			Predicted class					

## Data Availability

Data is available from the corresponding authors, upon reasonable request.

## Abbreviations

The following abbreviations are used in this manuscript:

AI	artificial intelligence
AUC	area under the curve
CAM	class activation mapping
CNN	convolutional neural network
DL	deep learning
LDCT	low-dose computed tomography
pCLE	confocal laser endomicroscopy
TL	transfer learning
